# Longer-term effects and underlying mechanisms of dietary intervention strategies on cognitive performance in adults with cognitive impairment

**DOI:** 10.1097/MCO.0000000000001133

**Published:** 2025-05-23

**Authors:** Tineke Degens, Tanja C. Adam, Ronald P. Mensink, Peter J. Joris

**Affiliations:** Department of Nutrition and Movement Sciences, NUTRIM Research Institute of Nutrition and Translational Research in Metabolism, Maastricht University Medical Center+, 6200 MD Maastricht, The Netherlands

**Keywords:** aging, cognitive impairment, cognitive performance, diet, randomized controlled trials

## Abstract

**Purpose of review:**

As global life expectancy increases, age-related neurodegenerative conditions such as dementia impose an increasing public health and socioeconomic burden. Maintaining a healthy lifestyle, particularly through a healthy diet, may reduce cognitive decline and support cognitive performance in aging populations. Despite increasing interest in dietary interventions as a strategy to enhance cognitive performance, research findings remain inconclusive. This narrative review aims to synthesize evidence on the longer-term effects (published February 2023–October 2024) and underlying mechanisms of dietary intervention strategies on cognitive performance in adults with preexisting cognitive impairment.

**Recent findings:**

Recent evidence from randomized controlled trials suggests that both single- and multifactor dietary interventions may improve one or more cognitive domains in aging adults with preexisting cognitive impairment. However, variability in intervention types, durations, and participant characteristics limits the ability to draw definitive conclusions.

**Summary:**

This review highlights the potential benefits of longer-term dietary interventions on cognitive performance in adults with cognitive impairment. It further integrates emerging mechanistic insights, suggesting that specific dietary components may exert neuroprotective effects primarily by reducing oxidative stress and neuroinflammation, and by enhancing brain vascular function. These mechanisms may promote neuroplasticity through the modulation of neurotrophic signaling pathways. Future research should focus on replicating these findings to validate their efficacy and the underlying mechanisms involved. This is essential for integrating dietary approaches into evidence-based guidelines for promoting long-term cognitive health.

## INTRODUCTION

Aging is characterized by a progressive decline in physiological and cognitive functions, which contributes to an increased risk of cognitive impairment and the development of neurodegenerative diseases, including dementia [1]. Growing evidence suggests that maintaining a healthy lifestyle, possibly through a healthy nutrient-rich diet, is crucial for reducing cognitive decline and for supporting cognitive performance in aging populations [2]. Cognitive performance, which reflects the ability to process information, retrieve memories, and make decisions, can be classified into distinct cognitive domains, including executive function, learning and memory, attention, psychomotor speed, and language-related skills [3]. These domains are differentially affected during aging. Given that diet may exert domain-specific effects on cognitive performance, it is crucial to evaluate cognitive outcomes across distinct domains. The present narrative review specifically examines randomized controlled trials (RCTs) involving adults with preexisting cognitive impairment, as they are more likely to benefit from dietary intervention strategies. By evaluating the longer-term effects of single- and multifactor dietary interventions, as well as whole-diet approaches, on cognitive performance, this review provides a comprehensive overview of recent research findings (February 2023–October 2024). The objective is to advance our understanding of effective dietary intervention strategies and their potential underlying mechanisms for improving cognitive performance in an at-risk population. 

**Box 1:**
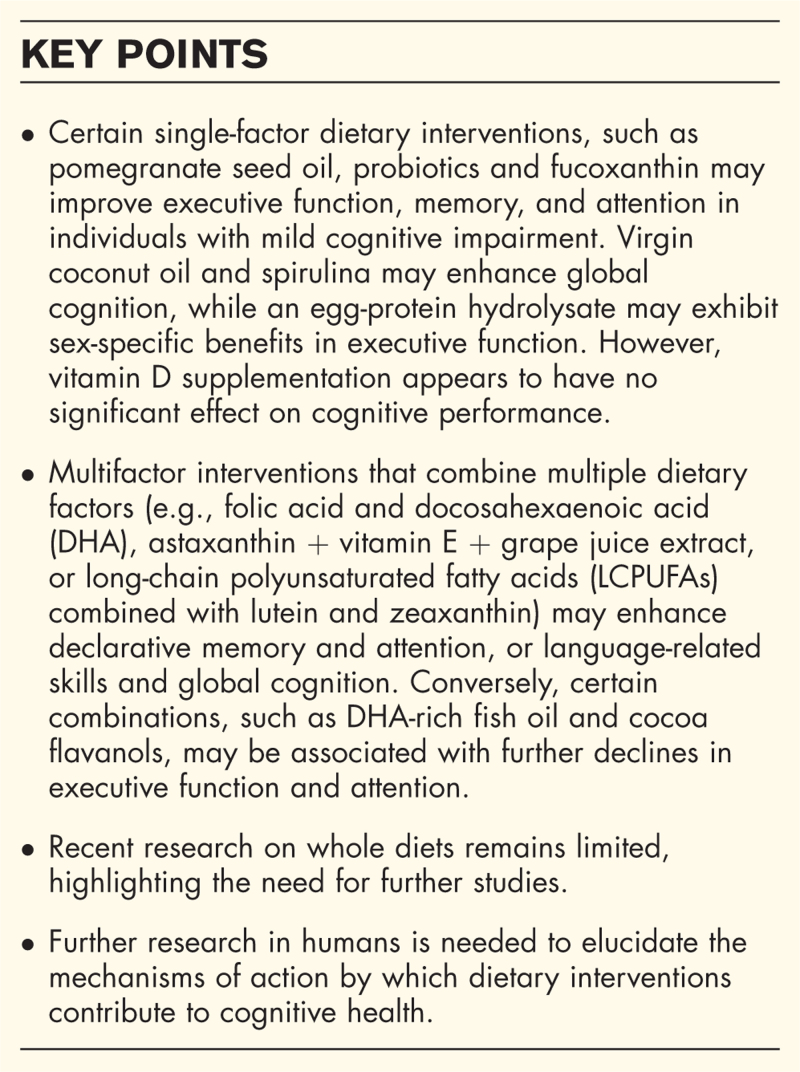
no caption available

## MATERIALS AND METHODS

### Search strategy

PubMed was searched to identify studies published in English between February 2023 and October 2024 using the primary search string: [(diet∗) AND (cognition OR brain)]. All retrieved articles were imported into EndNote, categorized, and screened for eligibility.

### Eligibility criteria

Eligible studies met the following criteria: (i) were RCTs; (ii) investigated longer-term dietary effects (≥7 days); (iii) included adults (≥18 years); (iv) involved participants with cognitive complaints, subjective (SCI) or mild cognitive impairment (MCI), or with (an increased risk for) dementia; and (v) assessed the effect on at least one cognitive-related outcome within a composite cognitive domain. Studies were excluded if they focused on combined lifestyle interventions.

### Data extraction

The following information was extracted separately for intervention and control groups from eligible studies and organized in Excel: (i) study characteristics; (ii) baseline characteristics; and (iii) cognitive (sub)domains. The study population was classified in cognitively impaired individuals, *i.e*. self-reported cognitive complaints, SCI, MCI, or (an increased risk for) dementia. Dietary interventions were grouped into three categories: (i) single-factor dietary interventions; (ii) multifactor dietary interventions; and (iii) whole diets.

## RESULTS

### Study characteristics

Thirteen double-blind RCTs were retrieved that focused on longer-term effects of dietary intervention strategies on cognitive performance in adults with preexisting cognitive impairments (Tables 1 and 2).

**Table 1:** Effects of dietary intervention strategies on cognitive (sub)domains in adults with preexisting cognitive impairments

					Executive function				Learning and memory			Attention and psychomotor speed			
First author (year)	Reference	Dietary intervention strategy	Dietary intervention	Study population	Overall	Working memory	Planning	Inhibition	Visuospatial	Declarative	Nondeclarative	Attentoin	Psychomotor speed	Language	Global cognition
Chatzikostopoulos (2024)	[4]	Single-factor	Pomegranate seed oil	MCI	↑					↑			↑		↑
Fei (2023)	[5]	Single-factor	Probiotics	Older adults with MCI	↑				↑	↑		↑			↑
Yoo (2024)	[6^▪▪^]	Single-factor	Microalgae extract	Older adults with memory complaints	↑	↑				↑		↑			
Montero-Odasso (2023)	[8^▪^]	Single-factor	Vitamin D	Older adults with MCI											X
Adams (2024)	[9^▪^]	Single-factor	Egg-protein hydrolysate	Adults with elevated SCD	↑^a^	↑^a^									
Tamtaji (2023)	[11]	Single-factor	Spirulina	Alzheimer's disease											↑
Fernando (2023)	[12]	Single-factor	Virgin coconut oil	Mild-to-moderate Alzheimer's disease											↑^b^
Vauzour (2023)	[13^▪^]	Multifactor	DHA-rich fish oil and cocoa flavanols	SCI or MCI	↓^c^							↓			
Lopresti (2024)	[14]	Multifactor	Astaxanthin, vitamin E, grape juice extract	Adults with self-reported cognitive complaints						↑					
Li (2024)	[15]	Multifactor	Folic acid and DHA	Older adults with MCI						↑^d^		↑		↑	↑
Sueyasu (2023)	[16]	Multifactor	LCPUFAs with lutein and zeaxanthin	Older adults with cognitive decline						↑					
Baltic (2024)	[17]	Multifactor	Dihydrogen-pyrroloquinoline quinone mixture	Older adults with MCI								↑			
Barnes (2023)	[19^▪▪^]	Whole diet	MIND diet	Older adults predisposed to dementia											X

a Women only.

b *APOE* ε4 carriers only.

c SCI only.

d Memory subdomain not specified.

**Table 2:** Baseline characteristics of randomized controlled trials investigating the effects of dietary intervention strategies in adults with preexisting cognitve impairment

							Gender (%)								
					Sample size		Intervention		Control		Age (years) mean±SD		BMI (kg/m^2^) mean±SD		
First author (year)	Study duration	Dietary intervention strategy	Intervention and Dose	Control and Dose	Intervention	Control	Male	Female	Male	Female	Intervention	Control	Intervention	Control	Ref.
Chatzikostopoulos (2024)	1 year	Single-factor	Pomegranate seed oil 5 drops	None (both groups followed MEDI diet	40	40	40	60	47.5	52.5	69.8 ± 3.1	69.3 ± 2.7	–	–	[4]
Fei (2023)	12 weeks	Single-factor	Probiotic mixture 2 g/d	Starch 2 g/d	20	20	47.6	52.4	52.4	47.6	76.40 ± 9.61	75.30 ± 9.75	22.9 ± 2.8	23.5 ± 1.7	[5]
Yoo (2024)	12 weeks	Single-factor	Microalgae extract: *Phaeodactylum tricornutum* 1100 mg/d	Maltodextrin 275 mg/d	22	21	59.1	40.9	47.6	52.4	64.3 ± 6.4	64.3 ± 6.2	26.7 ± 3.7	27.5 ± 4.3	[6^▪▪^]
Montero-Odasso (2023)	20 weeks	Single-factor	Vitamin D 10 000 IU dose 3x/week	Matched placebo	71	70	42.3	57.8	60	40	73.1 ± 6.8	72.75 ± 6.6	27.0 ± 4.9	28.4 ± 8.35	[8^▪^]
Adams (2024)	36 weeks	Single-factor	Egg-protein hydrolysate Newtricious-03 5.7 g/d	Maltodextrin 5.7 g/day	20	20	55	45	65	35	–	–	28.0 ± 2.8	28.3 ± 2.8	[9^▪^]
Tamtaji (2023)	12 weeks	Single-factor	Spirulina 500 mg 2x/d	Placebo 500 mg 2x/d	27	26	7.4	92.6	11.5	88.5	73.8 ± 9.9	76.9 ± 5.4	21.9 ± 2.2	23.3 ± 3.0	[11]
Fernando (2023)	24 weeks	Single-factor	Virgin coconut oil 15 mL 2x/d	Canola oil (equivalent amount)	43	41	25.6	74.4	43.9	56.1	-	-	33.3	-	[12]
Vauzour (2023)	1 year	Multifactor	EPA 0.4 g/d, DHA 1.1 g/d and cocoa flavanols 500 mg/d	80% palm oil and 20% corn oil; control chocolate drops without methylxanthines	125	121	45	55	41	59	66.0 ± 6.3	65.0 ± 6.7	27.2 ± 4.2	26.6 ± 4.5	[13^▪^]
Lopresti (2024)	12 weeks	Multifactor	Astaxanthin 9 mg/d, grape juice extract 250 mg/d, vitamin E 12 mg/d	Matched placebo	45	44	20	80	22	78	59.8 ± 1.0	58.9 ± 1.2	26.5 ± 0.6	26.8 ± 0.7	[14]
Li (2024)	1 year	Multifactor	Folic acid 800 μg/d and DHA 800 mg/d	Corn starch and soybean oil	70	70	37.2	62.8	41.4	58.6	66.0^a^	65.0^a^	25.4 ± 2.6	25.7 ± 2.8	[15]
Sueyasu (2023)	12 weeks	Multifactor	LCPUFAs (120 mg ARA, 300 mg DHA, 100 mg EPA) combined with 10 mg lutein and 2 mg zeaxanthin	Placebo	88	92	52	48	54.6	45.4	65.1 ± 0.6	65.1 ± 0.6	22.7 ± 0.3	22.8 ± 0.3	[16]
Baltic (2024)	6 weeks	Multifactor	Dihydrogen-pyrroloquinoline quinone mixture 100 mg 2x/d	Nondihydrogen producing magnesium bicarbonate with organic acids (equivalent amount)	18	16	11.1	88.9	25	75	72.4 ± 3.7	71.4 ± 4.0	26.4 ± 3.6	27.2 ± 3.5	[17]
Barnes (2023)	3 years	Whole diet	MIND diet with mild caloric restriction	Control diet with mild caloric restriction	301	303	34.9	65.1	35	65	70.4 ± 4.2	70.4 ± 4.2	33.8 ± 5.4	34.0 ± 6.5	[18]

a Median.

### Single-factor dietary interventions and cognitive performance

#### Executive function

Five studies investigated the effects of single-factor dietary interventions on executive function. Improvements in overall executive function were reported in individuals with MCI following one year of daily pomegranate seed oil supplementation and after 12 weeks of daily probiotic treatment containing 18 strains of *Lactobacillus*, *Bifidobacterium*, and *Lactococcus*[4,5]. Similarly, 12-weeks of daily fucoxanthin intake, a microalgae-derived compound, enhanced overall executive function in elderly individuals with perceived age-related memory impairment [6^▪▪^]. Vitamin D deficiency has been associated with compromised executive function and a substantially increased risk of dementia in cross-sectional studies [7]. However, no improvements were observed after 20 weeks of daily vitamin D supplementation in individuals with MCI, potentially because their baseline vitamin D concentrations were within the normal range [8^▪^]. Benefits may be more evident in individuals with severe vitamin D deficiency [7]. Of the four studies assessing working memory as a subdomain of executive function, only one study demonstrated improvements, reporting enhanced working memory and overall executive function after long-term fucoxanthin intake [6^▪▪^]. Another study observed that long-term consumption of an egg-protein hydrolysate Newtricious (NWT)-03 improved executive function and working memory in women with increased SCD, but not in men [9^▪^]. This sex-specific effect may be linked to the estrogen decline during menopause, which is thought to accelerate cognitive decline in specific brain regions [10].

#### Learning and memory

Four studies evaluated learning and memory, of which three reported positive effects on declarative memory. One of those found that a one-year daily intake of pomegranate seed oil improved verbal episodic memory in individuals with MCI [4]. Another study revealed that a 12-week daily intake of a probiotic mixture (>2 × 10^10^ active cultures) improved delayed recall and positively affected visuospatial memory in MCI individuals [5]. Additionally, enhancements were observed following long-term supplementation with *Phaeodactylum tricornutum*, a microalgae extract, in older adults with perceived cognitive and memory decline [6^▪▪^]. None of the studies assessed nondeclarative memory.

#### Attention and psychomotor speed

Four studies examined the effects of single-factor dietary interventions on attention and psychomotor speed. One study reported an improvement in processing speed among individuals with MCI following one year of pomegranate seed oil intake, likely attributed to its neuroprotective phenolic compounds [4]. Furthermore, daily probiotics supplementation for 12 weeks enhanced attention in older individuals with MCI [5]. Similarly, supplementation with *Phaeodactylum tricornutum* showed benefits for attention in older adults with perceived cognitive and memory decline [6^▪▪^].

#### Global cognition

Five studies assessed global cognition in individuals with cognitive impairment, four of which reported positive effects. A study revealed that 12 weeks of spirulina supplementation improved cognitive performance in patients with Alzheimer's disease [11]. Similarly, one year of pomegranate seed oil improved global cognition among individuals with MCI [4]. Moreover, 12 weeks of probiotic intake enhanced global cognition in older MCI adults [5]. Global cognition was also improved in *APOE ε4* carriers with Alzheimer's disease following 24 weeks of virgin coconut oil supplementation, but no effects were observed in *APOE ε4* noncarriers [12]. This lack of response in *APOE ε4* noncarriers may be due to their lower genetic susceptibility to Alzheimer's disease pathology. In contrast, 20 weeks of vitamin D supplementation resulted in no measurable effects on global cognition in individuals with MCI [8^▪^].

### Multifactor dietary interventions and cognitive performance

#### Executive function

One study examined the effects of multifactor dietary interventions on executive function. This study found a decline in executive function in individuals with SCI after one year of co-supplementation with DHA-rich fish oil, also providing eicosapentaenoic acid (EPA), combined with cocoa flavanols, whereas no effects were found in individuals with MCI [13^▪^].

#### Learning and memory

Five studies evaluated the effects on declarative memory. A 12-week study found that co-supplementation of astaxanthin, vitamin E, and grape juice extract improved episodic memory in adults with subjective cognitive complaints [14]. Another study reported that one year of daily folic acid and DHA co-supplementation improved memory in older adults with MCI, possibly by mitigating mechanisms underlying cognitive impairment [15]. Moreover, a 12-week study in older Japanese adults with cognitive decline (without dementia) found that supplementation with LCPUFAs, combined with lutein and zeaxanthin improved memory function [16]. In contrast, no effects were observed following six weeks of supplementation with a dihydrogen-pyrroloquinoline quinone (PQQ) mixture in elderly individuals with MCI [17], nor after one year of DHA-rich fish oil and cocoa flavanol co-supplementation [13^▪^].

#### Attention and psychomotor speed

Four studies investigated the effects of multifactor dietary interventions on attention, with two studies reporting positive effects. One study conducted in older adults with MCI observed that supplementation with folic acid and DHA for one year improved concentrated attention, characterized by a sustained focus [15]. Another study reported enhancements in orientation following 6 weeks of dihydrogen-PQQ supplementation in individuals with MCI [17]. In contrast, 1 year of DHA-rich fish oil and cocoa flavanol co-supplementation decreased alertness – a state of active attention – in individuals with memory complaints [13^▪^]. Additionally, a 12-week intervention combining astaxanthin, vitamin E, and grape juice extract had no effect on the accuracy of attention nor speed of information processing in adults with self-reported memory complaints [14].

#### Language-related skills

Two studies evaluated the effects of combined interventions on language-related skills. One trial found that 1 year daily supplementation with folic acid and DHA improved verbal comprehension in older individuals with MCI [15]. In contrast, six weeks of dihydrogen-PQQ intake showed no effect on language-related skills [17]. Given that neuronal remodeling and cognitive adaptations occur gradually, long-term supplementation periods may be required to achieve measurable improvements in language processing [18].

#### Global cognition

Two studies assessed the effects on global cognition, with both reporting positive results. One study reported that one year of folic acid and DHA supplementation enhanced global cognitive performance in older adults with MCI [15]. In contrast, 6 weeks of dihydrogen-PQQ intake did not affect global cognition in individuals with MCI [17].

### Whole diets and global cognition

#### Global cognition

Only one study has examined the effect of a whole diet on global cognition. Although this study did not fully meet the eligibility criteria, it provided a valuable comparison with other dietary strategies. This RCT evaluated the impact of the MIND (Mediterranean-DASH Intervention for Neurodegenerative Delay) diet with mild caloric restriction, compared to a control diet with equivalent caloric restriction, over a 3 year period in older adults with a family history of dementia. The MIND diet, designed to reduce dementia risk, combines elements of the Mediterranean and DASH (Dietary Approaches to Stop Hypertension) diets. The study found that adherence to the MIND diet did not improve global cognition [19^▪▪^]. This result is in contrast with evidence supporting the beneficial effects of individual dietary components on antioxidant and anti-inflammatory processes, which have been associated with slower cognitive decline and reduced neuropathological hallmarks of Alzheimer's disease [20^▪^,^21^]. The discrepancy may be attributed to the impact of caloric restriction on cognitive outcomes, which may have masked the beneficial effects of the MIND diet. It is known that caloric restriction may exert positive effects on systemic processes such as insulin sensitivity and chronic inflammation, which may also affect cognitive health, independent of diet [22]. Furthermore, although participants in this study had a genetic predisposition to dementia, they may have been cognitively healthy at baseline, potentially limiting the capacity to detect significant effects of the MIND diet on global cognitive function throughout the intervention.

### Underlying mechanisms

The brain is highly metabolically active and has a high oxygen demand, leading to the generation of reactive oxygen species (ROS). Oxidative stress, characterized by an imbalance between free radicals and antioxidants defenses, contributes to neuronal dysfunction, cell damage, and neuroinflammation [23]. The accumulation of oxidative stress is considered a key mechanism involved in the pathophysiology of neurodegenerative diseases, including Alzheimer's disease, as well as age-related cognitive decline [24]. Consequently, understanding diet-related mechanisms that protect the brain from oxidative stress is essential to develop targeted interventions to preserve cognitive health. This section provides a concise overview of key neuroprotective mechanisms, primarily based on *in vitro* and animal studies.

Dietary factors, including LCPUFAs such as DHA and EPA – found predominantly in fatty fish – may exert neuroprotective effects by reducing oxidative stress and neuroinflammation through their antioxidant and anti-inflammatory properties [15,16]. These effects may be facilitated by increased regional cerebral blood flow, as LCPUFAs beneficially affect brain vascular function, potentially slowing cognitive decline [25]. Moreover, co-supplementation with lutein and zeaxanthin – the predominant carotenoids in the human brain – may further enhance the neuroprotective effects of LCPUFAs by reducing oxidative stress and neuroinflammation [16,26]. In addition, other carotenoids such as fucoxanthin and astaxanthin have been reported to exhibit neuroprotective effects, potentially mediated through their antioxidant and anti-inflammatory properties [6^▪▪^,^14^]. These effects may influence Alzheimer's disease progression by modulating key pathological processes, including inhibition of beta-site amyloid precursor protein cleaving enzyme 1 and reduction of amyloid-β accumulation [27].

Folic acid, a B vitamin, plays a pivotal role in one-carbon metabolism, supporting DNA methylation and neurotransmitter synthesis. By regulating homocysteine metabolism, folic acid enhances cognitive performance and reduces the neurotoxic effects of elevated homocysteine concentrations, which sensitize neurons to oxidative stress and excitotoxicity [28,29]. Emerging evidence also highlights the crucial role of the microbiome-gut-brain axis in regulating cognitive health. Research suggests a potential link between gut microbiota imbalances, low-grade chronic inflammation, and the development of neurodegenerative diseases [30]. The gut microbiome may influence cognitive performance by producing neurotransmitters and neuroactive compounds, such as short-chain fatty acids that can cross the blood-brain barrier and may benefit brain regions involved in learning and memory [30,31]. Probiotics, which help modulate gut microbiota composition, have been shown to enhance cognitive performance by increasing the expression of tissue brain-derived neurotrophic factor (BDNF), particularly in brain regions such as the hippocampus and prefrontal cortex [5,32,33]. Furthermore, pomegranate seed oil, which is rich in polyphenolic compounds, has demonstrated neuroprotective effects by reducing pro-inflammatory cytokines, enhancing synaptic plasticity, and improving antioxidant enzyme activity [34]. Nonetheless, further research - especially in humans - is necessary to better understand the role of dietary interventions and their underlying mechanisms in supporting cognitive health.

## CONCLUSION

This review highlights the potential benefits of longer-term dietary interventions on cognitive performance in adults with cognitive impairment. Recent evidence from RCTs suggest that both single- and multifactor dietary interventions may improve key cognitive domains. However, variability in intervention types, durations, and participant characteristics limits definitive conclusions. Additionally, research on whole-diet approaches is limited. More rigorous, longer-term studies with standardized methods - such as objective cognitive testing and uniform dietary assessment - and diverse populations, including healthy individuals, are needed. This will not only conform the efficacy of specific dietary interventions but also facilitate the integrations of these approaches into evidence-based guidelines aimed at preserving cognitive performance in aging populations.

## Acknowledgements

*None.*

### Financial support and sponsorship

*The present review is not funded by any specific grant.*

### Conflicts of interest

*There are no conflicts of interest.*

## REFERENCES AND RECOMMENDED READING

Papers of particular interest, published within the annual period of review, have been highlighted as:

▪ of special interest
▪▪ of outstanding interest
